# Task-shifting in asthma and chronic obstructive pulmonary disease management: A review of the obstructive lung disease program

**DOI:** 10.12669/pjms.40.2(ICON).8945

**Published:** 2024-01

**Authors:** Madiha Siddiqui, Fizra Khan, Saima Saeed

**Affiliations:** 1 Madiha Siddiqui, FCPS The Indus Hospital & Health Network, Plot 3 & 3-A, Sector 47 Korangi Creek Road, Karachi, Pakistan; 2 Fizra Khan, BE The Indus Hospital & Health Network, Plot 3 & 3-A, Sector 47 Korangi Creek Road, Karachi, Pakistan; 3 Saima Saeed, FRCP The Indus Hospital & Health Network, Plot 3 & 3-A, Sector 47 Korangi Creek Road, Karachi, Pakistan

**Keywords:** Task shifting, Chronic respiratory diseases, Nurses, Low- middle-income countries, Primary care

## Abstract

**Objective:**

Task shifting, an approach to address physician shortage through redistribution of clinical tasks, may help address the high burden of chronic respiratory diseases like asthma and COPD. We aimed to measure its utility and impact in the Obstructive Lung Disease program (OLD).

**Methods:**

A retrospective, cross-sectional study was conducted at five integrated outpatient departments of Primary Care Program within Indus Hospital & Health Network, Pakistan, from January 2018 to March 2023. After a formative evaluation, registered nurses were trained as Lung Health Nurses (LHNs) to perform spirometry, collect Patient Reported Outcome Measures (PROMs) including Asthma Control Test (ACT), modified Medical Research Council (mMRC) dyspnea score and COPD Assessment Test (CAT), counsel on inhaler use and tobacco cessation, and refer to pulmonary rehabilitation (PR). Data was collected online contemporaneously on REDCap and later analyzed using Excel and STATA 14.

**Results:**

Pre-implementation, a monthly average of 126 asthmatics and 33 COPD patients visited primary care centers. Medical records of 147 OLD patients showed 8% received inhaler education, 3% completed ACT and 2% had mMRC documented. Implementation included capacity building of nine LHNs. Of 7427 referrals to the program, 86% underwent nurse-led assessments. LHNs performed spirometry (92%), PROMs assessments [ACT (89%), CAT (91%), mMRC (85%)], inhaler education (97%), tobacco cessation advice (85%) and made PR referrals (94%).

**Conclusion:**

Trained nurses can play a role in providing holistic and timely care for patients with CRDs and strengthen existing healthcare systems. Future directions may include expanding nurse clinical counselling roles through telehealth monitoring and home management.

## INTRODUCTION

Chronic respiratory diseases (CRDs) have impacted millions globally with an ever-escalating trend. The most common CRDs include asthma and Chronic Obstructive Pulmonary Disease (COPD). Symptoms include breathlessness, cough and wheeze, whilst definitive diagnosis is made by healthcare professionals (HCPs) using spirometry, a breath test for lung function assessment. To date, the major burden of asthma and COPD worldwide is in lower middle-income countries (LMICs).^1^ High morbidity, mortality and healthcare cost in these countries, due to uncontrolled disease and subsequent hospitalizations or emergency care visits, became more evident in the Covid-19 era.^2^ Numerous gaps have been highlighted in disease management and control. These include inequalities in accessing diagnostics and care, ineffective counselling on medication usage and adherence, improper self-management of disease and awareness of its chronicity.^3^

In Pakistan, prevalence has been estimated up to 12.1% for asthma and 13.8% for COPD.^4^,^5^ There are multiple risk factors including childhood respiratory infections, tobacco smoke, occupational exposures and air pollution (especially indoors). Effective responses to this may be multifactorial and include addressing the lack of an effectively trained and distributed healthcare workforce. Human resource shortage has been identified as a crisis by World Health Organization.^6^ Task shifting was successfully implemented in the management of Human Immunodeficiency Virus worldwide.^7^ In its global recommendations and guidelines, the WHO emphasized that effective and equitable task shifting mechanisms should not be restricted to infectious diseases, rather worked upon systematically in other essential health services as well. Task shifting involves strategic, regulated engagement of healthcare personnel (for example nurses, community healthcare workers) by transferring certain tasks to them from specialized HCPs after targeted, relevant training. Considering this an opportunity for addressing the burden of obstructive lung disease challenges in Pakistan, we utilized ‘task shifting’ in development of the Obstructive Lung Disease (OLD) program.

The OLD program at the Indus Hospital and Health Network (IHHN) focuses on the health care needs of individuals suffering from CRDs. It integrates diagnostics of susceptible patients seen within primary care and empowers them in managing their disease appropriately.^8^ Diagnosis includes using hand-held spirometry, whilst management requires several domains of counselling that can be time-consuming for busy physicians. Our study considers the feasibility of task shifting, employed in the OLD program to reduce the burden of spirometry and counselling of OLD patients from doctors to trained nurses. Here we report the impact on clinical task completion before and after implementation.

## METHODS

**Fig.1:**
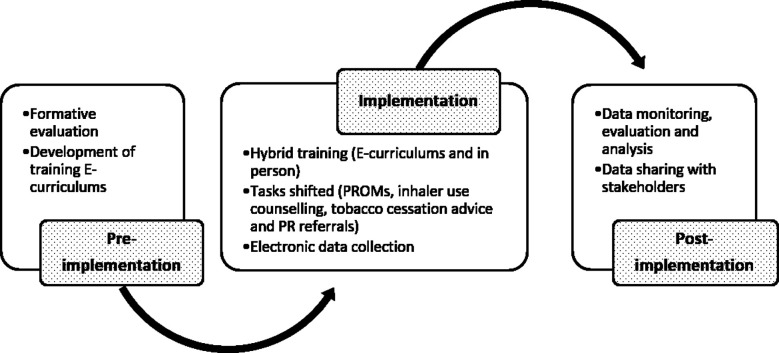
Stages of implementation of Task Shifting in Primary Car e Program.

A retrospective observational study was conducted in five old program centers set within the outpatient based Primary Care Program (PCP) of IHHN. The OLD program was initiated at The Indus Hospital, Korangi campus after stakeholder consultation with primary care and Pulmonology teams. This was replicated to allow expansion of services to Mushtaq Ahmed Gurmani Facility (Gurmani), Recep Tayyip Erdogan Hospital (Muzaffargarh), Al-Ghazi Trust Campus (Bhong) and Qarshi Foundation Naimat Saleem Trust (QFNST, Lahore). The processes involved in program implementation are mentioned below and have been summarized in Fig.1.

### Formative evaluation

A baseline assessment was performed to determine the number of asthma and COPD patients attending PCP clinics and whether documentation of management tasks was present. This data was retrieved from HMIS for 2018. It was filtered to select all ‘asthma’ and ‘COPD’ diagnosed cases presenting in clinics that year and average monthly calculated. In-depth review of medical notes was performed to identify documentation of relevant clinical tasks (mentioned below) for December 2018.

### Hybrid training

Registered nurses were specifically recruited and trained to become Lung Health Nurses (LHNs) through hybrid training, using e-curriculums and in-person sessions. E-curriculums were developed and run on Canvas, an online learning management system (Instructure, USA). It was later shifted to the IHHN Moodle platform enabling coordinated dissemination within the network. In-person training included shadowing a family physician or pulmonologist for at least two weeks, followed by directed training, by either a consultant pulmonologist or Nurse Trainer.

### Tasks shifted and data collection

Tasks shifted to trained LHNs were in the domains of spirometry performance, recording of Patient Reported Outcome Measures (PROMs) and education and counselling.

Spirometry tests were performed using handheld spirometers (MIR, Italy). Quality and results of reports were recorded, with reasons for which spirometry could not be performed. Quality A to C were considered good quality reports, based on analysis by spirometer’s internal software which checked technique through acceptability and repeatability of trials. This was used as a surrogate for the quality of patient counselling and LHN technical understanding.

PROMs included the modified Medical Research Council (mMRC) score to measure degree of chronic breathlessness, COPD Assessment Test (CAT) for control of COPD and the Asthma Control Test (ACT) for control in asthmatics were assessed contemporaneously by LHNs.

Patient education and counseling was completed at the time of patient encounter. Specific topics included (a) Inhaler education, whenever prescribed (b) referral to Pulmonary Rehabilitation if an mMRC of two and/or above was noted and (c) tobacco cessation advice for all current tobacco users.

Documentation of tasks reallocated to nurses were recorded as numerical values or checkboxes in an electronic form on REDCap, a secure web-based application for online database management.

### Data monitoring, evaluation and analysis

Data was monitored and evaluated routinely as part of OLD programmatic development. Patient referrals to the OLD program from the PCP were evaluated by the number of patients reaching LHN as a proportion of the total spirometry test requests by primary care physicians recorded in HMIS. Further monitoring and evaluation occurred in collaboration with LHNs and PCP staff monthly and impact summaries discussed quarterly. This data sharing with stakeholders streamlined activities, determined impact and addressed potential issues systematically.

### Ethics Committee approval

This was obtained from the Institutional Review Board (IHHN) with ID # IHHN_IRB_2022_12_020. Data was captured between January 2018 to March 2023 using pre-existing IHHN electronic management system, Health Management Information System (HMIS) and the bespoke REDCap tool applied to the OLD program. 

### Statistical Analysis

Data analysis using Excel and STATA was performed.

## RESULTS

### Formative evaluation

In 2018, 126 asthma and 33 COPD cases were clinically diagnosed in The Indus Hospital, Korangi campus PCP clinic on average every month. In the medical records of 147 asthma and COPD patients in December 2018, there were no spirometry referrals. Documentation of OLD related PROMs was 2% (mMRC), 3% (ACT in asthma medical records), 0% (CAT in COPD medical records) whilst inhaler technique review was mentioned in 8% cases. Tobacco cessation advice and referrals to physiotherapy department for management of breathlessness or physical activity were not documented.

### Hybrid training

Initially two registered nurses, and after expansion to other IHHN sites, an additional seven local registered nurses were trained. Completion rates of e-curriculums were 89% (Inhalers), 100% (Spirometry), 78% (Tobacco cessation), 56% (Asthma) and 56% (COPD). Asthma and COPD courses were aimed at primary care doctors and taken by LHNs after a period of experience within the OLD program, thus have ongoing enrolments.

### Tasks shifted and data collection

Out of 7427 referrals, a total of 6370 (86%) patients were reviewed on their first visit by LHNs. Descriptive statistics of tasks performed after diagnosis are shown in Table-I.

**Table-I:** Frequencies of task shifted at five integrated OLD sites from 2019 till 2023.

Tasks Shifted	Frequencies	

	N	%
Spirometry	5838 (6370)	92
Patient Reported Outcome Measures (PROMs):		
Asthma Control Test (ACT)	2594 (2923)	89
COPD Assessment Test (CAT)	1143 (1251)	91
modified Medical Research Council (mMRC) dyspnea scale	3539 (4174)	85
Inhaler education	2637 (2706)	97
Pulmonary Rehabilitation (PR) referrals	857 (910)	94
Tobacco cessation counselling	1388 (1733)	80

**Fig.2:**
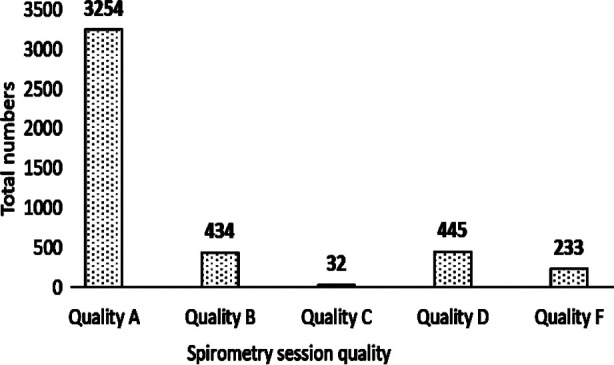
Number of spirometry quality achieved by nurses.

**Fig.3:**
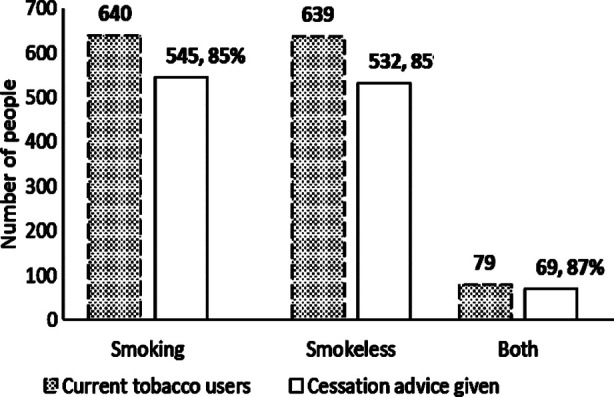
Number of current tobacco users and patients who received cessation advice.

A total of 4937 spirometry tests were conducted by LHNs of which 75% produced good quality reports. A breakdown of the spirometry quality is included in Fig-2. Spirometry tests were left incomplete in 1427 patients due to inability to meet a good quality test criterion. In 513 patients, spirometry was not attempted due to shortness of breath or cough (34%) or because there was no requirement for spirometry (34%). In other cases, the patient was not prepared for diagnostic spirometry having used an inhaler immediately prior (17%), technical errors (6%), language barrier (5%) or patient refusal (3%). Tobacco cessation advice was given to both smoked and smokeless tobacco users (Fig-3).

## DISCUSSION

Our study indicates that task shifting has a role in bridging gaps in patient care and management of CRDs in our setting as capacity building allows for tasks previously performed by doctors to be performed more frequently by nurses. An umbrella review highlighted the importance of training and other factors in implemention of task shifting as well as potential role of allied healthcare workers like nurses to meet healthcare demands.^9^ Another review focusing on articles from LMICs showed that tasks shifted to non physician healthcare workers in cancer screening and patient education were performed effectively and in certain cases even better than physicians.^10^ Other commonly occuring NCDs in LMICs have been managed successfully using this strategy, for example asthma education by lay-educators in Malawian children, cardiovascular disease (CVD) assessments and counselling by nurses in India for prevention of CVDs.^11^,^12^ In Pakistan, task shifting occurred in Covid home care and mental health interventions within communities by healthcare workers and peers.^13^,^14^

Worldwide, spirometry tests are conducted by trained healthcare professionals including doctors, registered respiratory therapists or registered cardiopulmonary function technologists. We chose to train nurses as they are more familiar with primary care settings for our integrated program and can easily multitask. An evaluation of spirometry for COPD screening in general practice concluded that spirometry tests by trained nurses produced results similar to trained doctors.^15^ Although we did not do a similar comparison, we utilized spirometry quality as a surrogate for training and have a high number of acceptable results. It must be noted, however, that spirometry performance and quality assessment also include patient comprehension which may contribute to these particular results.^16^

PROMs have been introduced as tools to standardize patient outcomes by supporting and directing individualized management plans.^17^,^18^ We successfully incorporated these into the OLD program, along with inhaler use education, referrals to pulmonary rehabilitation and tobacco cessation. Various studies demonstrate the advantages of this. In a study integrating nurse-led PROMs into primary care for multimorbidity (including asthma and COPD), feedback from both patients and nurses found this intervention to be feasible and highly acceptable.^19^ Nurse-led inhaler education led to improved medication compliance, adherence and reduction in hospital visits/admissions.^20^

Meanwhile, referrals to pulmonary rehabilitation are greater when performed holistically by nurses as opposed to general physicians.^21^ Finally, a tertiary care hospital in Pakistan conducted a nurse-led intervention to help cardiovascular and respiratory patients quit smoking, showing significantly reduced cigarette use.^22^ A systemic review found that nurse-led interventions in chronic diseases resulted in better health outcomes e.g., improvements in blood pressure, glycemic control, diet and physical activity levels, and reduced tobacco use. It also showed a high patient satisfaction level.^23^

Whilst our study did not record specific patient outcomes or satisfaction, it reports from the real-world experience of program implementation. There was communication and liaison through shared knowledge and ownership with the clinical and operational teams, as well as alignment and coordination of systems and activities, e.g., using electronic health record systems for monitoring and evaluation.

### Limitations

We were unable to formally assess clinical barriers and enablers for our intervention. Our formative evaluation, although defining the clinical burden, may not accurately describe the performance of clinical tasks if these were not documented in HMIS. Whilst didactic training of LHNs was considered essential for them to take on tasks that were shifted, there was ongoing on-the-job training after implementation that improved LHN performance as the program develops. Finally, the cost-effectiveness of task-shifting has not been evaluated here.

Despite these concerns, this study adds to the medical literature regarding the role of task shifting, particularly in Pakistan. Scaling within primary care can significantly impact diagnosis and management of CRDs, and other NCDs, in the country. Future work could involve enhancing the skillset of LHNs, expanding their role with newer tasks including monitoring and management using telehealth and community-based care.

## CONCLUSION

Our study demonstrates that task shifting in managing chronic respiratory diseases, by nurse led interventions, is both feasible and desirable in low resource settings such as Pakistan.

### Author`s Contributions:

**MS:** Conception, preparing the manuscript, designing of methodology and analysis, data interpretation, accountable for the accuracy and integrity of the study **FK:** Designing of data collection tools and acquisition of data, computations and analysis, manuscript drafting **SS:** Conception, manuscript editing, and revision for accuracy.
